# AKT overactivation can suppress DNA repair via p70S6 kinase-dependent downregulation of MRE11

**DOI:** 10.1038/onc.2017.340

**Published:** 2017-10-02

**Authors:** D Piscitello, D Varshney, S Lilla, M G Vizioli, C Reid, V Gorbunova, A Seluanov, D A Gillespie, P D Adams

**Affiliations:** 1 Beatson Institute for Cancer Research, Garscube Estate, Glasgow, UK; 2 Centre for Gene Regulation and Expression, School of Life Sciences, University of Dundee, Dundee, UK; 3 Epigenetics of Cancer and Ageing, University of Glasgow, Glasgow, UK; 4 Department of Biology, University of Rochester, Rochester, NY, USA; 5 Instituto de Tecnologías Biomédicas, Centro de Investigaciones Biomédicas de Canarias, Facultad de Medicina, Universidad de La Laguna, La Laguna, Tenerife, Spain

## Abstract

Deregulated AKT kinase activity due to PTEN deficiency in cancer cells contributes to oncogenesis by incompletely understood mechanisms. Here, we show that PTEN deletion in HCT116 and DLD1 colon carcinoma cells leads to suppression of CHK1 and CHK2 activation in response to irradiation, impaired G2 checkpoint proficiency and radiosensitization. These defects are associated with reduced expression of MRE11, RAD50 and NBS1, components of the apical MRE11/RAD50/NBS1 (MRN) DNA damage response complex. Consistent with reduced MRN complex function, PTEN-deficient cells fail to resect DNA double-strand breaks efficiently after irradiation and show greatly diminished proficiency for DNA repair via the error-free homologous recombination (HR) repair pathway. MRE11 is highly unstable in PTEN-deficient cells but stability can be significantly restored by inhibiting mTORC1 or p70S6 kinase (p70S6K), downstream kinases whose activities are stimulated by AKT, or by mutating a residue in MRE11 that we show is phosphorylated by p70S6K *in vitro*. In primary human fibroblasts, activated AKT suppresses MRN complex expression to escalate RAS-induced DNA damage and thereby reinforce oncogene-induced senescence. Taken together, our data demonstrate that deregulation of the PI3K-AKT/ mTORC1/ p70S6K pathways, an event frequently observed in cancer, exert profound effects on genome stability via MRE11 with potential implications for tumour initiation and therapy.

## Introduction

Phosphatase and tensin homologue deleted on chromosome 10 (PTEN) is a tumour suppressor gene found altered in multiple sporadic tumours.^1, 2^ Lesions in the PTEN gene occur at almost the same frequency as *TP53* alterations in certain tumour types.^3^ Its chief function is to antagonize phosphatidylinositol 3-kinase (PI3K) signalling, so that impaired PTEN function leads to unrestrained activation of its downstream signals and results in high levels of constitutively active AKT.^4^ AKT is key node on the PI3K pathway and controls the activation of the major signalling pathways for cell growth, survival and metabolism by phosphorylating many downstream signalling targets.^5^ The amplification of *PIK3CA* (the gene encoding for the p110α catalytic subunit of PI3K) also causes growth factor-independent constitutive activation of AKT, and is often found in ovarian and cervical cancers.^6, 7, 8^ mTOR complex 1(mTORC1) is a well known effector of activated AKT. AKT signals through direct phosphorylation of the TSC1/TSC2 complex to indirectly activate mTORC1.^9, 10^ A crucial effector of mTORC1 is 40S ribosomal protein S6 kinase (S6K).^11^ S6K directly regulates ribosome biogenesis, cell cycle progression, protein synthesis and metabolism.^12, 13^

The centrality of the genome to cell function, phenotype and viability means that challenges to genome stability and acquisition of genome instability have profound consequences for the cell. For example, primary non-transformed cells can undergo senescence if they incur irreparable DNA damage. Senescence is an irreversible growth arrest associated with morphological and gene expression changes.^14, 15^ Expression of the oncogenic form of RAS (HRASG12V) can lead to oncogene-induced senescence (OIS) due to accumulation of unrepaired damaged DNA caused by unscheduled DNA synthesis.^16, 17^ DNA damage-activated OIS poses a potent barrier to tumourigenesis,^14, 15^ and cells that escape or bypass OIS are at risk of progression to cancer.

In contrast to primary non-transformed cells, cancer cells often possess an unstable genome that leads to gross genetic alterations, clonal evolution and tumour heterogeneity.^18, 19^ The cellular DNA damage response (DDR) has a crucial role in the maintenance of genomic stability. Mutations in the DDR pathway allow the survival and proliferation of cells with genomic abnormalities, promoting oncogenic transformation and therefore tumourigenesis. However, inherent defects in DNA repair processes also provide an important therapeutic opportunity. For instance, tumours deficient for *BRCA1/2* genes are highly sensitive to interstrand DNA crosslinking agents, such as cisplatin and carboplatin, and to a new class of anti-cancer agents called poly (ADP-ribose) polymerase (PARP) inhibitors.^20, 21, 22^

Inactivation of PTEN has been linked to genome instability in cancer. For example, an early report showed that lack of PTEN initiates genome instability through mislocalization of CHK1.^23^ Another report showed that PTEN confers centromeric stability and suppression of DNA double-strand breaks, in part though control of RAD51 function.^24^ Potentially contributing to genome instability in PTEN-deficient cells, several lines of evidence have shown that the PI3K/PTEN/AKT pathway has a role in modulating cell-cycle checkpoint activation and DNA repair. High levels of AKT can inhibit homologous recombination (HR) repair by suppressing the formation of BRCA1 and Rad51 foci, specifically after exposure to Irradiation (IR) in breast cancer.^25^ Overactivated AKT or PTEN loss can also overcome the DNA damage-induced G2 cell cycle checkpoint and Chk1 activation upon exposure to genotoxic stresses.^23, 26, 27, 28, 29^ Thus, neoplastic cells expressing constitutively active AKT can avoid apoptosis and checkpoint-dependent cell cycle arrest, and accumulate potential cancer-causing mutations due to suppression of HR and reliance on error-prone NHEJ.

Given the potent suppressor activity of PTEN, we set out to discover additional mechanisms by which inactivation of PTEN promotes genome instability. We show that increased AKT-mTOR-S6K activity upon loss of PTEN leads to phosphorylation and degradation of MRE11 nuclease and impairs the DNA damage response in colorectal carcinoma cells. However, in non-transformed primary fibroblasts, elevated AKT activity, suppression of DNA repair and accumulation of DNA damage lead to a consolidation of RAS-induced senescence. Therefore, we propose a new mechanism by which loss of PTEN and consequent activation of the PI3K-AKT-mTORC1-S6K1 signalling pathway impairs DNA repair by downregulation of MRE11. In primary cells, this accumulated DNA damage can reinforce tumour suppression, but in cancer cells can promote genome instability.

## Results

### PTEN deficiency suppresses DNA damage signalling via MRN complex hypomorphism

**Figure 1:**
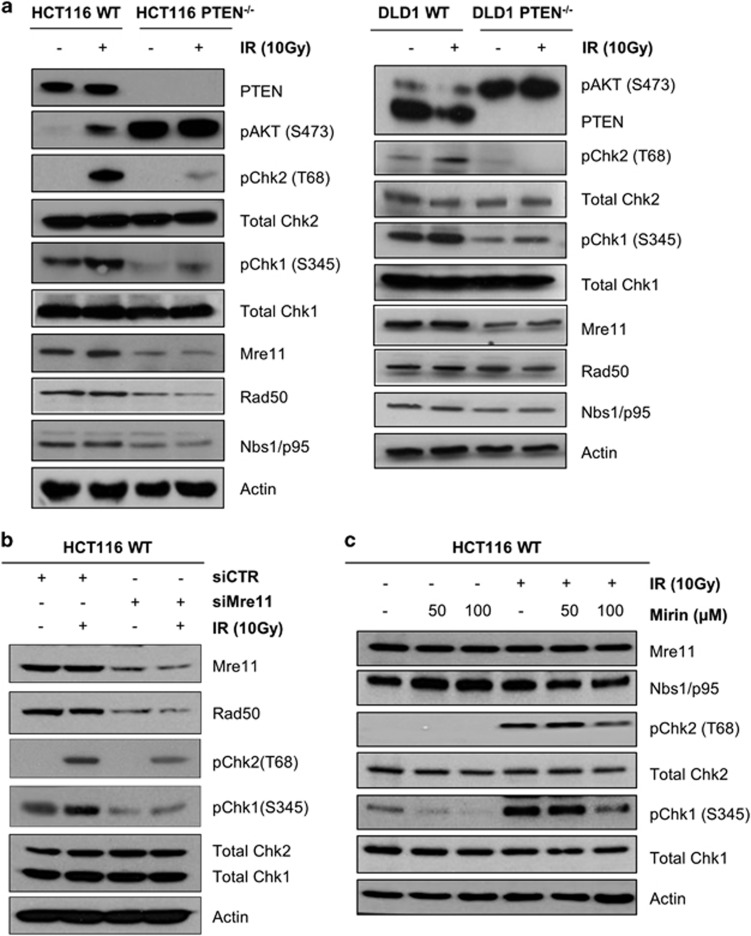
PTEN deficiency suppresses DNA damage signalling via MRN complex hypomorphism. (**a**) Western blots of indicated proteins were performed on whole-cell extracts from HCT116 and DLD1 (WT or PTEN^−/−^) cells 1h post irradiation with 10 Gy. Actin provides the loading control. (**b**) Western blot analysis was performed on whole-cell extracts from HCT116 WT cells 48 h post transfection with 200 nm smart pool siRNA against *MRE11* or non-targeting siRNA pool and 1 h post irradiation with 10 Gy. Actin provides the loading control. (**c**) Western blot analysis was performed on whole-cell extracts from HCT116 WT cells, generated 1 h after exposure to 10 Gy irradiation and pre-treatment with 50 and 100 μm Mirin for 40 min. Actin provides the loading control.

To investigate the impact of PTEN deficiency on DNA damage signalling, we first compared irradiation-induced activation of CHK1 and CHK2 in HCT116 colon carcinoma cells and an isogenic sister cell line in which *PTEN* was ablated by gene targeting. As expected, HCT116 PTEN^−/−^ cells exhibit very high basal levels of active AKT phosphorylated at serine 473 (S473) compared with the parental HCT116 cell line owing to the absence of PTEN expression (Figure 1a, left). Western blot analysis 1 h post irradiation revealed that activation of CHK1 (phosphorylated at S345) and CHK2 (phosphorylated at T68) was significantly impaired in HCT116 PTEN^−/−^ cells compared with the control. Importantly, diminished activation of CHK1 and CHK2 was also observed in isogenically matched DLD1 and DLD1 PTEN^−/−^ cells, indicating that this phenomenon is not confined to HCT116 (Figure 1a, right).

The MRE11/RAD50/NBS1 (MRN) complex is a key regulator of DNA damage signalling that is required both for optimal activation of ATM, the upstream regulator of CHK2, and for activation of ATR, the upstream regulator of CHK1. Remarkably, we found that the basal expression level of both MRE11 and its partner protein RAD50 were significantly diminished in HCT116 PTEN^−/−^ cells compared with the control (Figure 1a, left). Previous publications have suggested that HCT116 cells contain MRE11 mRNA-lacking exons 5–7, causing expression of a truncated non-functional protein.^30^ However, RT–PCR on cDNA from our HCT116 WT and PTEN^−/−^ cells did not reveal the presence of the mutated cDNA (Supplementary Figure 1A). In addition, we only detect the full-length protein in our western blot assays. In agreement with the HCT116, a similar decrease in the basal expression of MRE11 was observed in DLD1 PTEN^−/−^ cells (Figure 1a, right). However, RAD50 was not as markedly reduced as in HCT116 PTEN^−/−^ cells, suggesting that additional factors govern stability of these two proteins. We then investigated the effect of siRNA-mediated depletion of MRE11 on DNA damage signalling. Reduced MRE11 expression impaired activation of both CHK1 and CHK2, indicating that the lower level seen in PTEN^−/−^ cells is indeed limiting for optimal DNA damage signalling (Figure 1b). We also pre-treated wild-type HCT116 cells with Mirin, a small molecule inhibitor of MRE11 catalytic activity, and found that 100 μm Mirin also impaired activation of CHK1 and CHK2 after irradiation (Figure 1c). Taken together, these observations indicate that diminished levels of MRN contribute to the attenuation of CHK1 and CHK2 activation in PTEN-deficient cells after irradiation.

### Checkpoint proficiency and DNA repair is attenuated in the absence of PTEN

**Figure 2:**
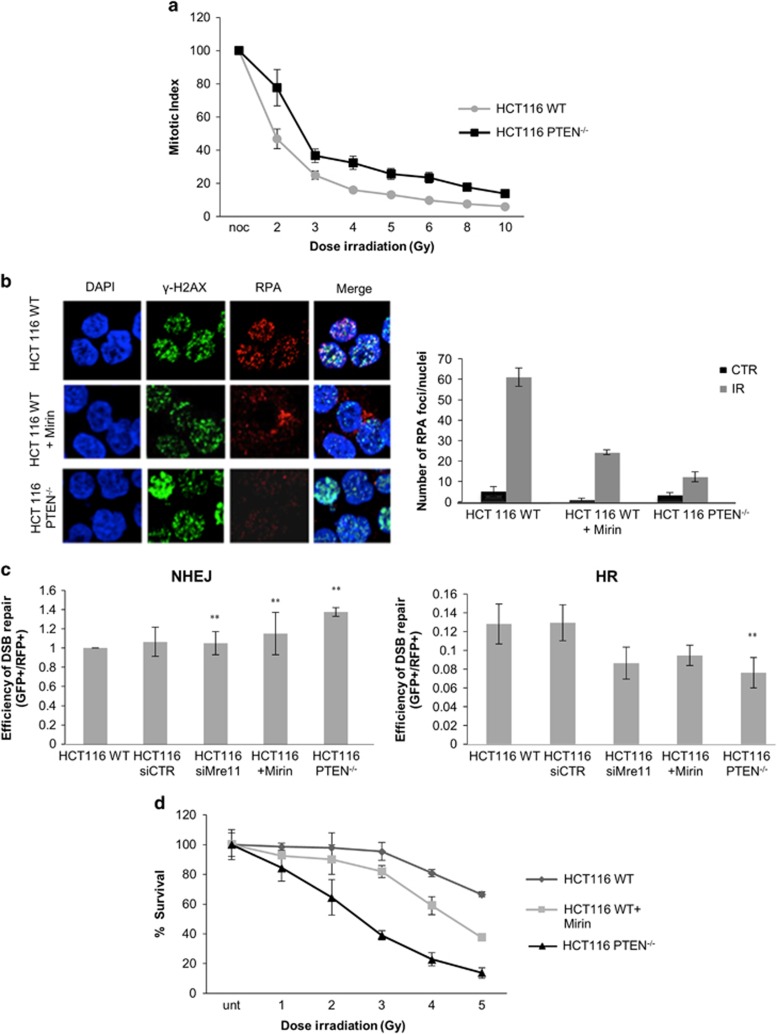
Checkpoint proficiency and DNA repair is attenuated in the absence of PTEN. (**a**) FACS analysis measuring phopho-histone H3 levels and PI incorporation in replicate cultures of HCT116 WT and PTEN^−/−^ cells with or without prior exposure to different doses of IR and incubation with nocodazole for 9 h. FACS data present the mitotic index (ratio of cells in M-phase positive for phosphorylated H3 over total number of cells × 100). The data are normalized to the nocodazole (noc) sample not exposed to IR and average±S.D. of *n*=3 are plotted. (**b**) Immunofluorescence analysis of phosphorylated H2Ax (γ-H2AX) and RPA foci in HCT116 WT with or without pre-treatment with Mirin, and in PTEN^−/−^ cells 4 h after exposure to 10 Gy IR. Quantification of the number of RPA foci per nuclei. Error bars show S.D. for three independent biological replicates. (**c**) Frequency of NHEJ and HR was analysed with two independent NHEJ and HR constructs in HCT116 WT and PTEN^−/−^ cell lines. The WT cell line was either pre-transfected with control siRNA or siRNA pool against *MRE11* or pre-treated with 15 μm Mirin 40 min prior to transfection with linearized constructs. The ratio of GFP+/DsRed+ cells was used to measure repair efficiency and is presented as a percentage of the untreated WT control. Error bars show s.e.m. for three independent biological replicates. ** denote *P*<0.01 as calculated by Student’s *t*-test. (**d**) Survival curves plotted with the number of colonies determined by the GelCount (Oxford Optronix, Abingdon, UK). % Survival was calculated relative to the cells untreated with IR. Error bars show s.d. for three independent biological replicates.

Activation of CHK1 and CHK2 is required for cell cycle arrest in G2 in response to DNA damage. Therefore, we investigated whether this checkpoint was altered in the absence of PTEN. To this end, we treated HCT116 and HCT116 PTEN^−/−^ cells with nocodazole for 9 h, with or without prior irradiation at increasing doses, and performed cell cycle analysis by fluorescence-activated cell sorting (FACS) of cells stained with propidium iodide and an antibody to histone H3 phosphorylated at serine 10 (the latter a marker of mitosis). This analysis revealed that the mitotic index, that is, percentage of cells in M-phase, was considerably higher in HCT116 PTEN^−/−^ cells compared with wild-type at all doses tested, indicating that fewer cells succeeded in arresting in G2 after irradiation-induced damage (Figure 2a).

Cell cycle arrest in G2 is thought to provide cells with an opportunity to repair DNA damage, particularly using the error-free mechanism of HR. As MRN has a key role in initiating HR by promoting the formation of single-stranded DNA (ssDNA) by strand resection, we considered that this process might be attenuated in the absence of PTEN function. The loss of PTEN does not lead to alterations in the cell cycle profile of HCT116 cells (Supplementary Figure 1B). We therefore compared the amount and distribution of ssDNA formed in wild-type and HCT116 PTEN^−/−^ cells after irradiation using replication protein A (RPA) focus formation as a surrogate marker. HCT116 PTEN^−/−^ cells show marked reduction in RPA foci compared with wild-type, indicating a failure to form ssDNA efficiently (Figure 2b). In addition, a similar effect was observed in parental HCT116 cells pre-treated with 100 μm Mirin prior to irradiation, arguing that the formation of ssDNA/RPA foci also depends on MRE11 catalytic activity (Figure 2b).

To measure proficiency for DNA repair directly, we transfected HCT116 wild-type and PTEN^−/−^ cells with plasmids containing modified green fluorescent protein (GFP) coding sequences that permit the efficiency of repair mediated by HR or non-homologous end-joining (NHEJ) to be measured directly by flow cytometry as described previously.^31^ Remarkably, this analysis revealed that DNA repair mediated by HR was much less efficient in HCT116 PTEN^−/−^ cells compared with control, whereas proficiency for NHEJ was increased (Figure 2c). Importantly, the efficiency of HR in parental HCT116 cells could be reduced both by prior siRNA-mediated depletion of MRE11 or pre-treatment with Mirin, indicating that a normal basal level of MRE11 expression and catalytic activity are necessary for optimal HR under these conditions.

Defects in DNA repair frequently confer increased sensitivity to radiation and other DNA damaging agents. Consistent with the observed deficit in HR capacity, HCT116 PTEN^−/−^ cells were very much more sensitive to radiation than wild-type parental cells over a wide range of doses when tested in a clonogenic survival assay (Figure 2d). Clonogenic cell survival was also compromised when cells were treated with Mirin during the irradiation and recovery period, although the scale of this effect was less than the difference between parental and HCT116 cells HCT116 PTEN^−/−^ (Figure 2d).

### MRE11 is rapidly degraded in PTEN-deficient cells

**Figure 3:**
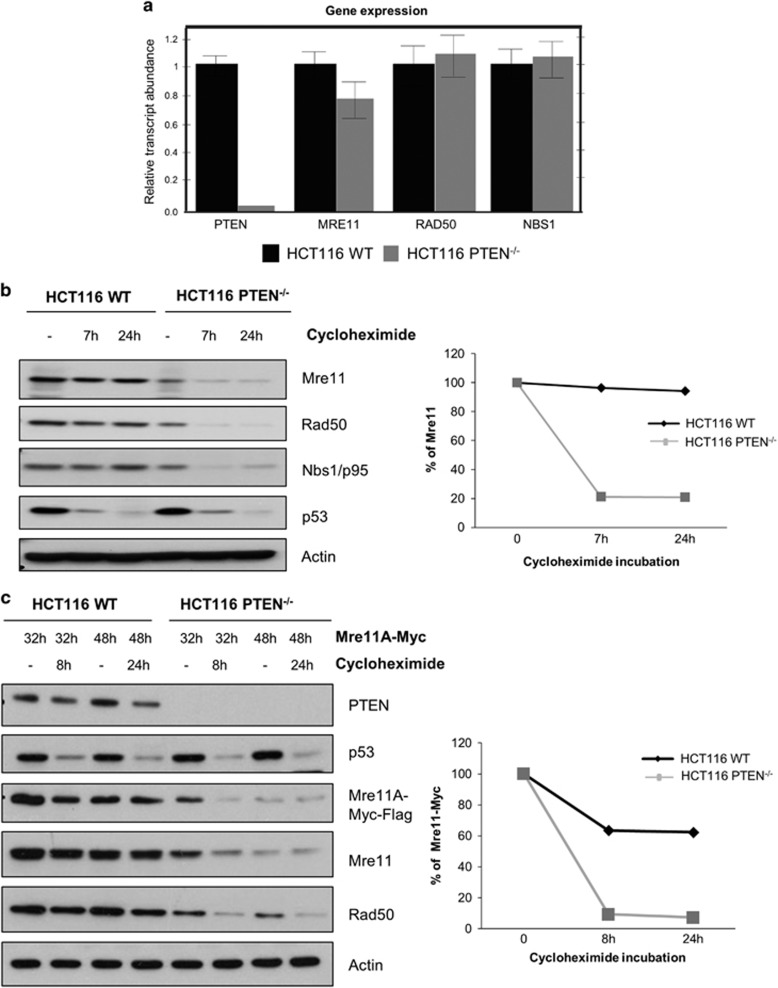
MRE11 is rapidly degraded in PTEN-deficient HCT116 cells. (**a**) RT–qPCR analysis of *MRE11, RAD50* and *NBS1* mRNAs in HCT116 WT and PTEN^−/−^ cells. Data were normalized to *ACTIN* mRNA and are presented relative to the WT signal. The error bars represent the s.d. of three independent biological replicates. (**b**) Western blot analysis of HCT116 WT and PTEN^−/−^ cells following treatment with cycloheximide for 7 and 24 h. p53 provides the positive control and actin provides the loading control. The graph represents the quantification of the MRE11 western blot analysis using ImageJ. (**c**) Western blot of HCT116 WT and PTEN null cells 32 h post transfection of ectopic MRE11A with or without cycloheximide treatment for 8 and 24 h. The graph represents the quantification of the MRE11A-Myc-Flag western blot signal by ImageJ.

The diminished levels of MRN components seen in HCT116 PTEN^−/−^ cells compared with parental HCT116 could result from decreased transcription of genes encoding these proteins, more rapid protein degradation, or a combination of both. RT–qPCR analysis revealed no significant difference in the levels of mRNAs encoding MRE11, RAD50 and NBS1/p95. However, as expected, PTEN mRNA was essentially undetectable in the genetically engineered PTEN knockout line (Figure 3a). To evaluate protein stability, we treated HCT116 and HCT116 PTEN^−/−^ cells with cycloheximide for 7 and 24 h. MRE11, RAD50 and NBS1/p95 were degraded more rapidly in HCT116 PTEN^−/−^ cells compared with parental HCT116 (Figure 3b). In striking contrast, p53 was equally labile in both cell types.

We also examined the stability of exogenous MRE11 transfected into parental and PTEN-deficient HCT116 cells. Cells transfected with a vector encoding Myc-Flag-tagged human MRE11 for 32 or 48 h, were treated with cycloheximide or vehicle for a further 8 or 24 h, respectively. This analysis demonstrated that exogenous MRE11 did not accumulate to the same level in HCT116 PTEN^−/−^ as parental HCT116 cells (Figure 3c). Consistent with this, when the cells were challenged with cycloheximide, we observed that the exogenous, tagged MRE11 protein was degraded more rapidly in PTEN-deficient than parental HCT116 cells. As before, p53 was equally labile in both cell types (Figure 3c).

### mTORC1 and p70S6 kinase signalling promotes MRE11 degradation

**Figure 4:**
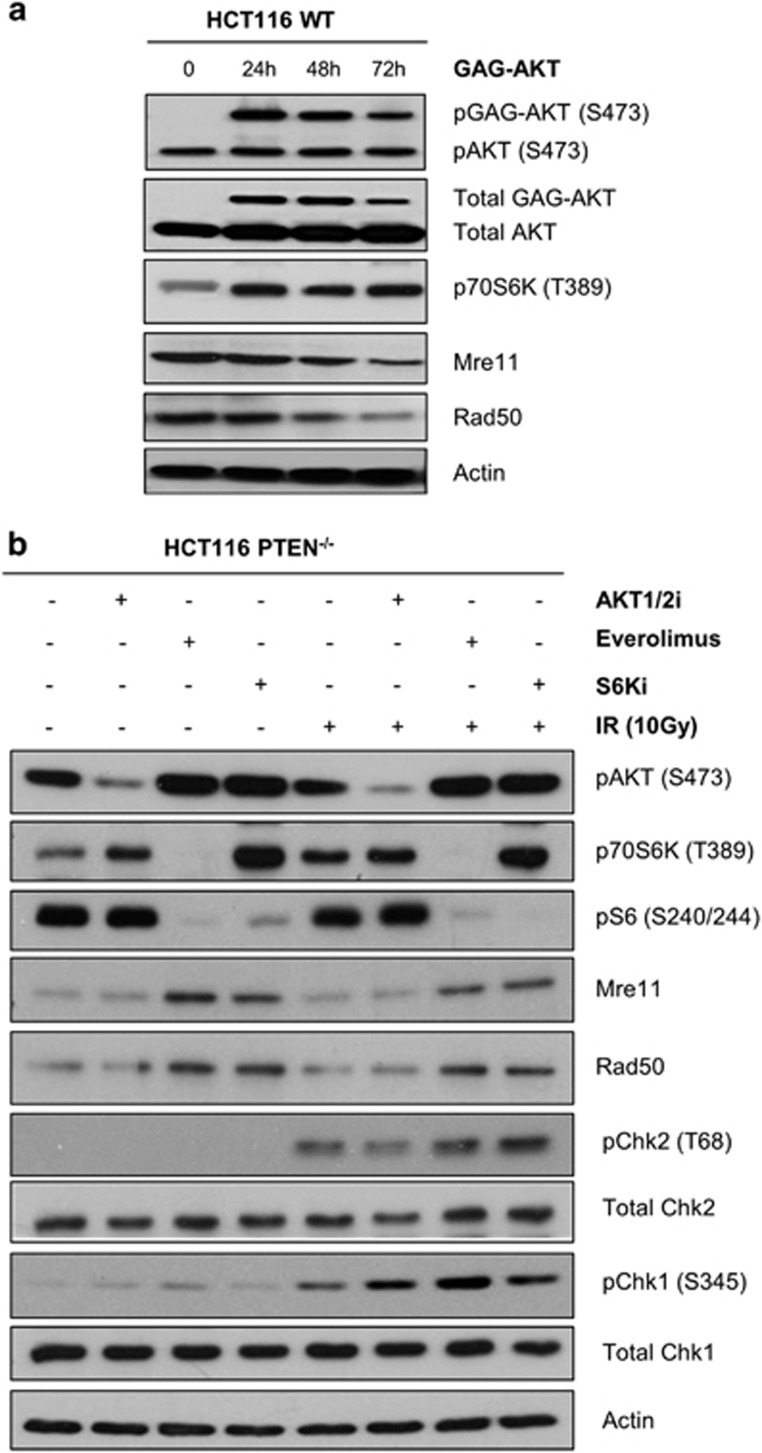
MRE11 suppression is stimulated by mTORC1 and S6 kinase signalling. (**a**) Western blot analysis of HCT116 WT cells transiently transfected with GAG-AKT and lysed at the indicated time-points post transfection. Actin provides the loading control. (**b**) Western blot analysis of HCT116 PTEN^−/−^ cells treated for 72 h with different inhibitors targeting various kinases within the AKT/mTOR/S6K pathway. Seventy-two hours post treatment, cells were exposed to IR and 1 h after were analysed. Actin provides the loading control.

Inactivation of PTEN elevates the AKT activity (Figure 1a). To determine whether MRE11 stability could be diminished by increasing the basal level of AKT activity in parental HCT116 cells, we overexpressed GAG-AKT, a constitutively active form that is resistant to inhibition by PTEN. This augmented the overall level of active, S473-phosphorylated AKT two- to threefold after 24 h (Figure 4a). Importantly, after 48 and 72 h of elevated AKT activity, there was a several fold decrease in the basal levels of MRE11 and RAD50, indicating that expression of a constitutively active form of AKT can mimic the effect of PTEN deletion.

We next asked whether the converse manipulations, namely chemical inhibition of AKT, or the downstream kinases mTORC1 and p70S6K, could rescue the diminished level of MRE11 and impaired DNA damage signalling seen in PTEN-deficient cells. To this end, we treated HCT116 PTEN^−/−^ cells with inhibitors of AKT (AKT1/2i), mTORC1 (Everolimus) or p70S6K (S6K1i) for 72 h and then evaluated the expression levels of MRE11, and activation of CHK1 and CHK2 either with or without irradiation.

Surprisingly, inhibition of AKT alone had little or no effect on MRE11 and RAD50 levels or CHK2 activation after irradiation, although there was a small increase in CHK1 activation compared with control (Figure 4b). However, inhibition of AKT had little or no effect on the activity of the downstream kinase mTORC1, monitored using antibodies specific for its substrate p70S6K, or on the activity of p70S6K itself, monitored using antibodies specific for phosphorylated ribosomal protein S6 (Figure 4b). The reason for this is currently unclear, as it is well-established that AKT stimulates the activity of mTORC1 and p70S6K, although it may be related to the existence of feedback loops within the AKT-mTOR-p70S6K pathway. Regardless, the failure of AKT inhibition to affect MRE11, RAD50 and CHK2 is likely due to its failure to inhibit these key downstream effectors, mTORC1 and p70S6K.

Consistent with this interpretation, we observed that direct inhibition of mTORC1 or p70S6K led to a pronounced increase in MRE11 and RAD50 levels and significant rescue of CHK1 and CHK2 activation after irradiation. As expected, inhibition of mTORC1 greatly inhibited both p70S6K and S6 phosphorylation, whereas inhibition of p70S6K diminished S6 phosphorylation without impairing mTORC1 activity (as judged by p70S6K phosphorylation). Taken together, these data implicate mTORC1 and p70S6K activity in the control of MRE11 stability and checkpoint proficiency. By the simplest model, mTORC1 exerts its effects on MRE11 and RAD50 via p70S6K.

### P70S6 kinase phosphorylates MRE11 to promote degradation

**Figure 5:**
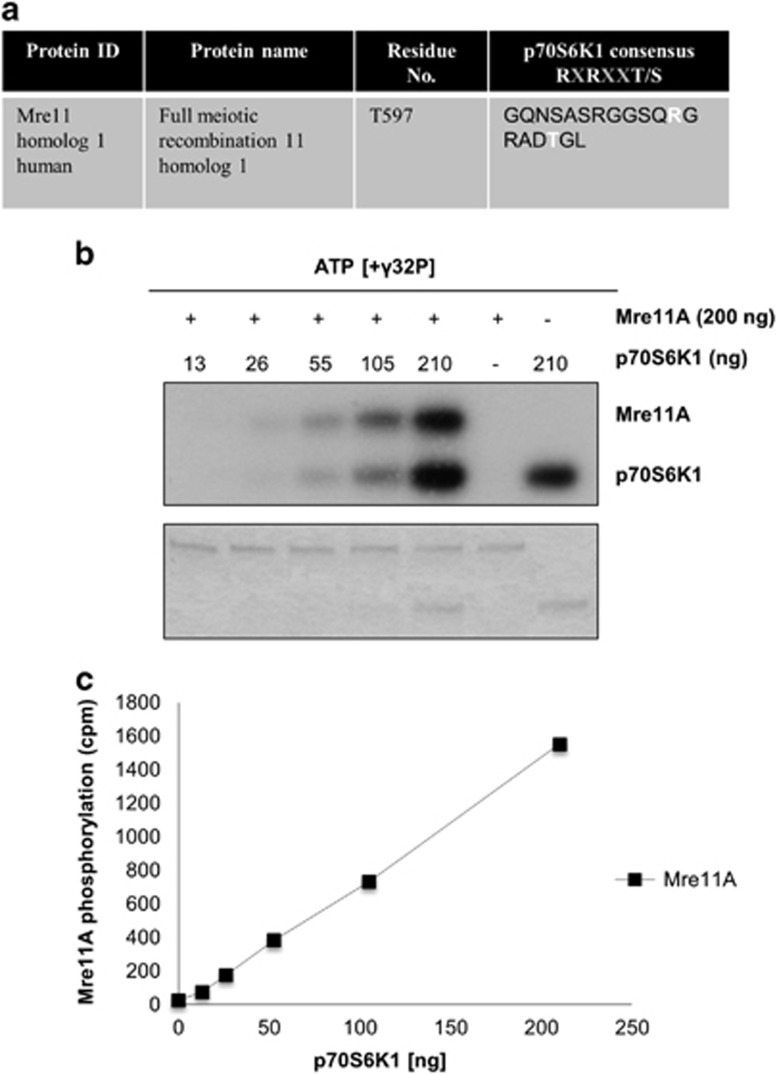
S6K1 phosphorylates MRE11A *in vitro*. (**a**) Sequence analysis of MRE11A reveals the presence of an S6K1 consensus sequence. The analysis identified a site on T597. (**b**) SDS–PAGE performed on recombinant Myc-MRE11A incubated with γ32P-ATP and varying amounts of recombinant S6K1. The lower panel shows Coomassie staining. (**c**) Scintillation counts of MRE11 bands cut from the SDS–PAGE gel quantifying 32P incorporation into MRE11A.

**Figure 6:**
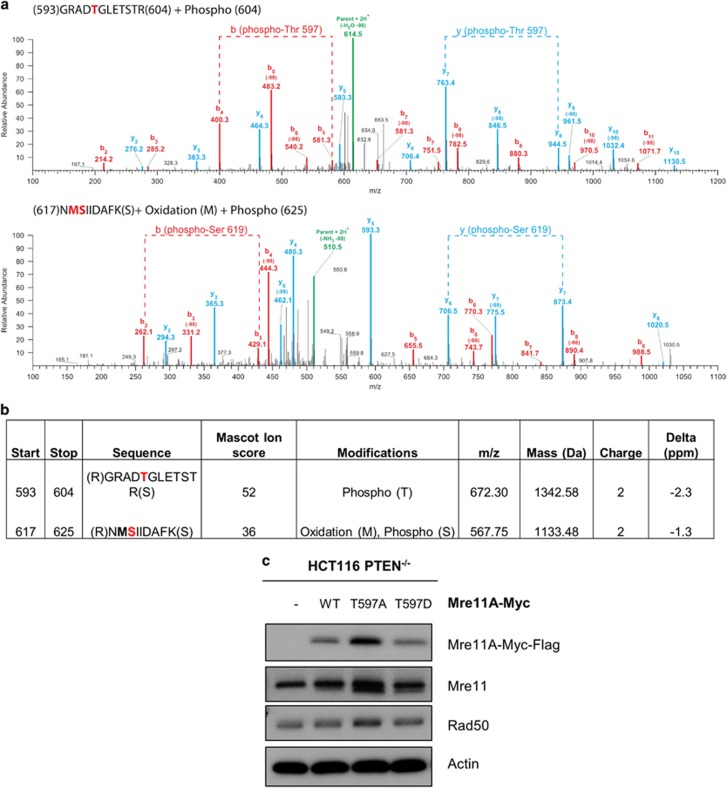
MRE11 degradation is mediated by S6K1 phosphorylation on T597. (**a**) Positive ion MS/MS spectra, of tryptic phosphopeptides (593–604) and (617–625) of MRE11. The *b* and *y* ion series are indicated in blue and red, respectively. Dashed lines indicates *b* and *y* ion fragments used to infer phosphorylation positions. (**b**) Summary of **a**. (**c**) Western blot of HCT116 PTEN null cells expressing ectopic MRE11A WT or MRE11A T597A or MRE11A T597D 24 h post transfection. Actin provides the loading control.

*In silico* analysis of MRE11 identified a threonine residue in position 597 (T597) as a potential target of p70S6K phosphorylation, identical to the p70S6K consensus target site RXRXXT/S (Figure 5a). To determine whether MRE11 could serve as a direct substrate for p70S6K, we performed an *in vitro* kinase assay using purified recombinant MRE11 and p70S6K proteins. We observed that increasing amounts of p70S6K catalysed increased incorporation of ^32^P into a fixed amount (200ng) of recombinant MRE11A as observed by autoradiography and scintillation counting following SDS–PAGE (Figures 5b and c). The extent of MRE11 substrate phosphorylation was similar to that of p70S6K auto-phosphorylation, suggesting that the reaction was efficient. To identify the sites of *in vitro* phosphorylation of MRE11, we analysed the phosphorylated protein by mass spectrometry. This analysis confirmed that T597 was a prominent site of phosphorylation, with lesser modification of serine 619 (S619: Figures 6a and b). Although we have not directly shown in this study that MRE11 is phopshorylated on T597 in PTEN null cells, recently published data have shown that the expression of Myr-AKT in cells induces Mre11 phosphorylation at T597.^32^ Altogether, these data are consistent with the idea that in PTEN null cells with activated AKT, S6K1 phosphorylates MRE11 on T597 to direct its degradation.

Previous experiments showed that exogenous MRE11 was also significantly less stable in HCT116 PTEN^−/−^ cells than wild-type cells (Figure 3c). To determine whether phosphorylation of T597 might have a role in controlling MRE11 stability in this cellular background, we generated mutants of MRE11 where T597 was replaced by a non-phosphorylatable alanine residue (MRE11 T597A) or by a phospho-mimetic aspartic acid residue (MRE11 T597D) by site-directed mutagenesis. When transfected into HCT116 PTEN^−/−^ cells, MRE11 T597A accumulated to significantly higher levels than either MRE11 wild-type or MRE11 T597D (Figure 6c). Although phosphorylation of T597 should be confirmed *in vivo*, these results are at least consistent with phosphorylation of T597 having a role in promoting MRE11 degradation.

### Activated AKT reinforces RAS-induced senescence via MRN complex hypomorphism

Aside from ionising radiation, oncogene activation has also been established as a powerful trigger of DNA damage and DNA damage signalling. In primary human cells, expression of oncogenic HRAS leads to an initial burst of cell proliferation associated with a high level of DNA replication stress and DNA damage, leading eventually to cellular senescence.^33^ The detrimental effect of activated AKT on MRE11 expression, and DNA repair proficiency would modify cellular response to replication stress, consequently altering OIS. To investigate this possibility, early passage IMR90 human fibroblasts were transduced with retroviruses encoding HRASG12V or activated, myristoylated AKT (Myr-AKT) or both together.

**Figure 7:**
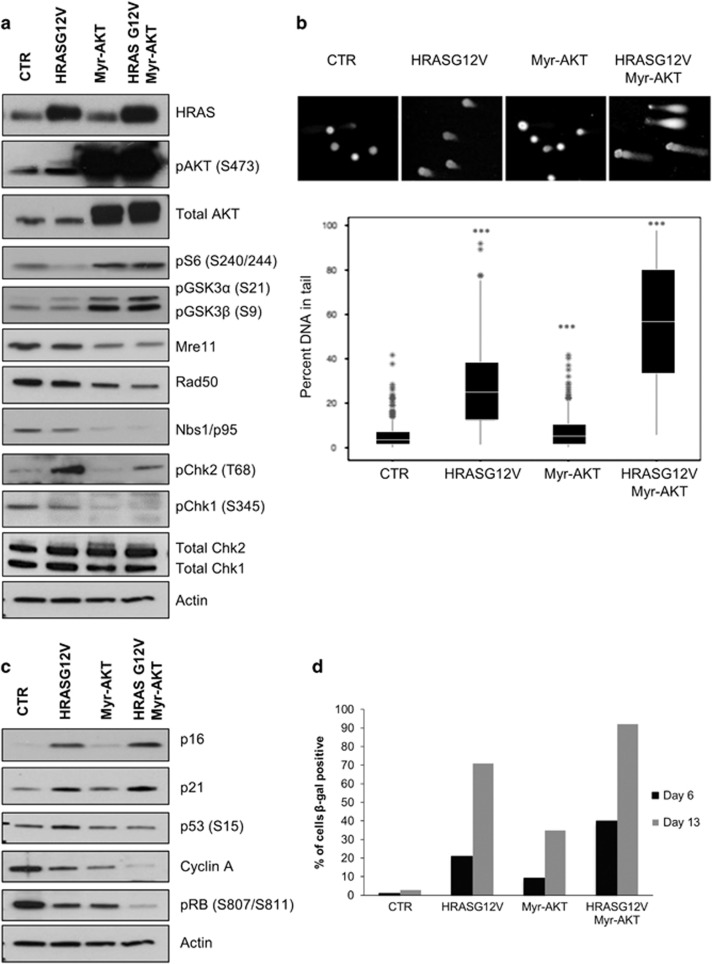
Activated AKT pathway enhances RAS-induced senescence by inhibition of MRN complex. (**a**, **c**) Western blot analysis on IMR90 cells 12 days post infection with retroviruses expressing HRASG12V, Myr-AKT, HRASG12V/Myr-AKT or control virus (CTR). Actin provides a loading control. (**b**, top) Immunofluorescence images obtained following an alkaline comet assay with IMR90 cells 12 days post infection with CTR, HRASG12V, Myr-AKT or HRASG12V and Myr-AKT retroviruses. (**b**, bottom) Total DNA damage (sum of single and double DNA breaks) was measured as percentage of DNA migrated in the tail of the comet over the total signal from the head and tail. Error bars show S.D. for three independent biological replicates. *** denotes *P*<0.001 as calculated by Student’s *t*-test. Data were quantified by using ImageJ OpenComet, and it was derived from three independent biological replicates. (**c**) Western blot analysis on IMR90 cells 12 days post infection with retroviruses expressing HRASG12V, Myr-AKT, HRASG12V/Myr-AKT or control virus (CTR). Actin provides a loading control. (**d**) Quantification of SA β-galactosidase-positive cells plotted as a percentage of total cells. The quantification was performed on the average of 100 cells per sample.

Expression of exogenous HRASG12V and Myr-AKT in freshly transduced IMR90 cultures was confirmed by Western blotting. The activity of Myr-AKT was evident through increased phosphorylation of AKT on serine 473, of the direct AKT targets GSK3α/β, and of the downstream mTORC1-p70S6K target, ribosomal protein S6 (Figure 7a). As expected, HRASG12V expression caused activation of DNA damage signalling, as judged by phosphorylation CHK2 at T68 (Figure 7a). When expressed alone, Myr-AKT did not induce DNA damage signalling as judged by CHK2 T68 phosphorylation, although the basal level of CHK1 S345 phosphorylation was suppressed (Figure 7a). As observed in HCT116 cells, expression of active Myr-AKT in IMR90 cells resulted in reduced expression of MRE11, RAD50 and NBS1 (Figure 7a). Moreover, when combined with HRASG12V, active Myr-AKT inhibited Ras-induced activation of CHK2 and suppressed the basal level of active CHK1, consistent either with a defect in DNA damage signalling or mitigation of HRASG12V-induced DNA damage.

To evaluate the impact of HRASG12V and Myr-AKT on genomic integrity directly, an alkaline comet assay was performed. As expected, cells transduced with HRASG12V showed a higher level of DNA damage than control cultures, whereas cells expressing Myr-AKT alone did not (Figure 7b). Importantly, cells expressing HRASG12V and Myr-AKT showed a significant increase (*P*<0.001) in comet tail length when compared with HRASG12V only cells, ruling out the possibility that Myr-AKT suppresses DNA damage signalling by mitigating HRASG12V-induced DNA damage (Figure 7b).

We next asked whether expression of Myr-AKT modified HRASG12V-induced cell senescence. IMR90 cell cultures were transduced with HRASG12V, Myr-AKT or both together, and analysed by western blotting for key senescence markers after 2 weeks in culture. Replicate dishes were stained for expression of senescence-associated β-galactosidase (SA β-gal) activity 6 and 13 days after transduction. As shown in Figure 7c, this analysis revealed that expression of HRASG12V-induced cell senescence, as judged by an increase in the expression of the cyclin-dependent kinase inhibitors p16 and p21, an increase in p53 serine 15 (S15) phosphorylation, and a marked decrease in the expression of cyclin A and phosphorylation of the retinoblastoma tumour suppressor (pRb) at serines 807 and 811 (S807/ 811) compared with control cultures. That these changes were associated with senescence was confirmed by a large increase in the proportion of cells expressing SA β-gal (Figure 7d).

In marked contrast, expression of Myr-AKT alone did not affect p16 and p21 expression, although cyclin A expression and pRb S807/ 811 phosphorylation were diminished compared with control cells. Consistent with this, SA β-gal expression was also increased in Myr-AKT-expressing cultures, although to a lesser extent than cells expressing HRASG12V alone (Figure 7d). Strikingly, cells transduced with both HRASG12V and Myr-AKT showed both upregulation of p16 and p21 and a more severe suppression of cyclin A expression and pRb S807/811 phosphorylation than cells expressing either oncogene alone (Figure 7c). Cultures expressing both HRASG12V and Myr-AKT also contained a higher proportion of cells expressing SA β-gal (Figure 7d).

The treatment of ER-RAS expressing cells with Mirin leads to increased p16 and p21 levels when compared with untreated cells (Supplementary Figure 2A). Mirin also causes a greater percentage of cells to arrest in G1 phase (Supplementary Figure 2B). This suggests that reduced MRE11 expression is likely to contribute to the observed synergy between AKT and HRASG12V. We conclude, based on these assays, that co-expression of Myr-AKT enhances DNA damage induced by HRASG12V and reinforces the senescence phenotype.

## Discussion

Here we have characterized a novel mechanism by which inactivation of PTEN impairs cell cycle checkpoints and DNA repair, and so promotes genome instability. Specifically, our evidence supports a model whereby elevated AKT-mTORC1 signalling in PTEN-deficient cells drives S6K-mediated phosphorylation and hence destabilization of MRE11, a catalytic nuclease whose activity is critical for DNA end resection and HR-mediated repair.^34, 35^ This occurs in at least two cell models, WT and PTEN null HCT116 and DLD1 cells. At least in HCT116 cells, this is associated with downregulation of the whole MRN complex, impaired DNA end resection and suppression of error-free HR repair. Interestingly, in DLD1 cells, loss of PTEN downregulates abundance of catalytic subunit MRE11, but has a more modest effect on MRE11’s non-catalytic partner proteins, RAD50 and NBS1. The reason for the difference between MRE11 and NBS1 in the two cell lines is not known, but could reflect genetic or epigenetic variations, which in turn modulate expression of NBS1 and MRE11. Regardless, as MRE11 is the catalytic exonuclease, its downregulation alone presumably impairs MRE11-mediated end resection. Of course, there are gaps in this study. For example, due to the instability of the wild-type protein in PTEN-deficient cells, we have not shown whether ectopic expression of MRE11 rescues any defects in PTEN-deficient cells. Owing to the same reason, it has proved difficult to map phosphorylation sites on endogenous MRE11 to confirm its phosphorylation on T597. Despite such relative weaknesses, our study provides a substantial body of data in support of the proposed mechanism.

In addition to unleashing unrestrained activity of mitogenic growth-promoting signalling pathways, inactivation of PTEN has long been linked to genome instability in cancer. Here, we have linked PTEN to preservation of genome stability though a novel mechanism that depends on its control of the mitogenic kinase signalling cascade, AKT-mTORC1-S6K impinging on the MRN complex. This coupling of mitogenic signalling to genome instability is intriguing. At first, it seems counterintuitive that a high level of mitogenic and nutrient signalling driving cell growth and proliferation should be associated with decreased DNA repair and increased genome instability. Yet, in fact, elevated mTOR activity is associated with accumulation of diverse types of molecular and cellular damage in many different biological systems and species. Hence, increased mTOR activity is tightly linked to decreased cellular and organismal longevity, conserved across evolution.^36^ In this light, the link between high mTOR activity and genome instability is not so surprising. Of course, our findings in cancer cells and differentiated fibroblasts might not apply to all other cell types. Indeed, in murine hematopoietic stem and progenitor cells, mTOR promotes the DDR and suppression of DNA damage by upregulation of FANCD2.^37^ Nevertheless, our study shows that at least colon cancer cells lacking PTEN are consequently deficient in DNA damage and repair. This might confer an advantage for the tumour by making the genome more plastic and evolvable. However, it may also have therapeutic implications. Cancers deficient in HR show promise as targets of precision medicine approaches that exploit this weakness in the cancer’s defence. Our study underscores previous studies that have linked loss of PTEN to defective HR repair.^38^

In this study, we also showed that inactivation of PTEN function recapitulated through activation of AKT cooperates with an activated oncogene to drive robust OIS. This finding is seemingly at odds with a previous study of Peeper and coworkers^39^ showing that inactivation of PTEN promotes bypass of BRAFV600E-induced senescence. On the other hand, the observations reported in this MS are consistent with others previously published from our lab.^40^ In this prior study, we showed that although inactivation of PTEN and activation of AKT bypassed some features of OIS, these interventions failed to overcome oncogene-induced proliferation arrest, an observation recapitulated here. DNA damage and DNA damage signalling are major drivers of cellular senescence-associated proliferation arrest.^33^ Hence, the increased damaged DNA load and DNA damage signalling in cells lacking PTEN is expected to acutely promote senescence-associated proliferation arrest, as we observed here. However, in a mouse model of pancreatic cancer, we also found that inactivation of PTEN cooperates with activated RASG12D to escape senescence and promote pancreatic cancer, in an mTOR dependent manner. As we noted previously,^40^ in this *in vivo* model, inactivation of PTEN might cooperate with RASG12D to escape senescence and promote pancreatic cancer via cooperation with additional acquired and selected mutations. This current study supports the notion that inactivation of PTEN can, possibly by destabilization of MRE11 and suppression of HR, promote the accumulation of such additional cooperating genetic rearrangements and mutations.

In sum, this study sheds light on the molecular mechanism by which inactivation of PTEN promotes tumorigenesis and may confer sensitivity to a specific class of anti-cancer therapeutic.

## Materials and methods

### Cell culture, transfections and chemical inhibitors

HCT116 WT, HCT116 PTEN^−/−^, DLD1 and DLD1 PTEN^−/−^ (gift from Todd Waldman), Phoenix-AMPHO embryonic kidney cells (SD-3443, ATCC)(used for the generation of helper-free ecotropic and amphotropic retroviruses) cells were grown in DMEM supplemented with 10% FBS, 2 mm L-glutamine, 50 U/ml penicillin and 50 μg/ml streptomycin at 37 **°**C. Human foetal lung primary fibroblasts IMR90 were obtained from Coriell Cell Repositories. They were cultured in DMEM supplemented with 20% FBS, 2 mm l-glutamine, 50 U/ml penicillin and 50 μg/ml streptomycin at 37 **°**C and 3% O_2_.

### Cells were irradiated using the Xstrahl RS225 unit

HCT116 WT cells were pre-treated for 40 mins with 50 and 100 μm of Mirin (M9948 Sigma, Schnelldorf, Germany) before experimental treatment as appropriate. HCT116 PTEN^−/−^ were pre-treated for 72 h with 2 μm of AKT1/2 inhibitor (A6730 Sigma), 500 nm of Everolimus (07741 Sigma) and 10 μm of S6K1 inhibitor (PF4708671 Tocris Bioscence, Abingdon, UK) before experimental treatment as appropriate.

MRE11A and non-targeting siRNA (L009271, D001810 Dharmacon, Lafayette, CO, USA) were transfected into HCT116 WT cells using Lipofectamine 2000 (Invitrogen, Thermo Fisher Scientific, Carlsbad, CA, USA). All siRNA transfection were performed with a final concentration of 200 nm for 48 h.

### Immunocytochemistry

Coverslips were pre-treated with extraction buffer (25 mm HEPES pH 7.4, 50 mm NaCl, 1 mm EDTA, 3 mm MgCl_2_, 300 mm sucrose, 0.5% Triton-X100) as described previously.^41^ The cells were fixed with 4% of paraformaldehyde for 15 min at room temperature, permeabilize with 0.5% Triton-X and incubated with blocking buffer (3% of BSA in PBS) for 30 min. The cells were then incubated with 1:200 dilution of primary antibody overnight and 1:200 secondary antibody for 1 h. The cells were then stained with DAPI, and images acquired with a × 60 oil immersion lens on a NikonA1R laser scanning confocal microscope (Tokyo, Japan). The number of foci were counted manually by using ImageJ software (National Institute of Health, Bethesda, MD, USA).

### Flow cytometry

Cells were fixed in ice-cold 70% ethanol in PBS and kept at 4 °C for 30 min. For the measurement of mitotic index cells were stained with anti-phospho histone H3 (pS10 H3) and propidium iodide, and analysed at FACScan flow cytometer as described previously.^42^ For cell proliferation analysis cells were labelled with 25 μm BrdU, and then fixed and stained using anti-BrdU antibody as described previously.^43^

The NHEJ and HR reporter vectors were obtained from Vera Gorbunova’s lab.^31^ In the extrachromosomal assay, 0.5 μg of linearized NHEJ or 2 μg of HR reporter construct were co-trasfected with 0.1 μg of pDs-Red2-N1 as transfection control, and analysed after 24 h by FACScan (Becton Dickinson FACScan Analyser, Walpole, MA, USA). Cells transfected with 1 μg of GFP-expressing plasmid, 1 μg of pDs-Red2-N1 and 1 μg of a control plasmid were used as calibration controls for the FACS prior to the analysis. The efficiency of DNA DSB repair was calculated as the ratio of GFP+ to DsRed+ cells.

### Gene expression analysis

SuperScript III reverse transcriptase (Invitrogen, Thermo Fisher Scientific) was used as per manufacturers instructions with 0.4 μg of RNA and Hexanucleotide Mix (Roche, Sigma, Schnelldorf, Germany). RT–PCR was performed using the C1000TM Thermal Cycler (Bio-Rad) using PerfeCTaTM SYBR Green FastMixTM (Quanta Bioscience, Beverly, MA, USA) and 0.25 μm of primers in Supplementary Table 1. The average of the ΔΔC(*t*) of duplicate samples was calculated using actin as a lading control. Overall average and s.d. values were calculated from three independent experiments.

### Recombinant protein purification

pGEX4T1 purchased from Novagen (Madison, WI, USA) and MRE11A cloned using In-Fusion HD (Clontech, Mountain View, CA, USA). Recombinant GST-MRE11A was expressed in BL21 *E. coli* plus RIL strain from pET156P vector. Bacteria were cultured in Terrific Broth at 37 °C to an *A*_600_ of 1.2 and protein expression was induced with 0.4 mm IPTG overnight at 16 °C. Cells were harvested by centrifugation, washed in ice-cold PBS and lysed in 50 mm Tris-Cl pH 7.5, 250 mm NaCl, 0.5 mm EDTA, 0.5 mm EGTA, 0.5% Triton-X100, 1 mm DTT, 20 μg/Ml leupeptin, 1 mm pefabloc, 0.5 mm TCEP by sonication. GST-MRE11A was purified using glutathione affinity resin, quantified by *A*_280_ with a Nanodrop and analysed by SDS–PAGE prior to use.

### Western blotting

Whole-cell lysates were resolved by SDS–PAGE, immobilized to PVDF, and subjected to western blotting as previously described.^44^ Antibodies used are listed in Supplementary Table 2.

### Kinase assay

A range of 13–210 ng of kinase and the 200 ng of substrate were incubated at 30 °C for 30 min along with 2 mm DTT, 20 mm Tris, 1 mm EGTA, 1 mm MgCl_2_ and 1 μl of 2 μCi/μl [γ-32P]. 4 × NuPAGELDS (Invitrogen, Thermo Fisher Scientific) sample buffer was added to the samples, then boiled at 95 °C for 5 min and resolved on a SDS–PAGE gel, which were coomassie stained. Signal was visualized by autoradiography. The proteins bands of interest were then excised and 32P incorporation was quantified by Cerenkov counting in a scintillation counter.

### Alkaline comet assay analysis

Alkaline comet assay was perfomed using the Comet assay kit (Trevigen, Gaithersburg, MD, USA) and performed as described by manufacturer. DNA was stained using SYBR Gold (Invitrogen, Thermo Fisher Scientific) and visualised by fluorescent microscopy. Images were analysed by using OpenComet plug-in in ImageJ, and 100 cells were counted for each treatment.

### β-galactosidase staining

Cells were seeded onto glass coverslips prior the onset of senescence. SA β-gal staining was performed as previously described^45^ and visualised using conventional bright field microscopy.

### Proteolytic digestion of proteins ‘in gel’

The region containing MRE11A was excised from gel and digested according to a previously described procedure.^46^

### Mass spectrometry

The tryptic digest obtained was separated by nanoscale C18 reverse-phase liquid chromatography using an EASY-nLC II coupled to a Linear Trap Quadrupole-Orbitrap Velos Thermo Fisher Scientific, Carlsbad, CA, USA.

The mass spectrometer was used in data-dependent mode, the top ten most intense ions from each survey scan were fragmented in the linear ion trap using collision-induced dissociation enabling the ‘multistage activation’ option.

### Data analysis

Raw data obtained were processed with Raw2MSN,^47^ and analysed using Mascot (Matrix Science, version 2.4.1, Boston, MA, USA), querying both: *Homo sapiens* UniProt database (release 2014_01, UniProt consortium, Cambridge, UK, restricted to, 20273 entries)^48^ and an in-house database containing common proteomic contaminants and the sequence of MRE11 construct.

Cysteine carbamidomethylation was specified as fixed modification, and variable modifications were allowed for acetylation of protein N terminus, oxidation of methionine, and phosphorylation of serine and threonine and tyrosine.

Scaffold (version 4.3.2, Proteome Software, Portland, OR, USA) was used to validate MS/MS-based peptide and protein identifications, and peptide false discovery rate was 0.9%.^49^
