# Supplementary figures and images for: AKT overactivation can suppress DNA repair via p70S6 kinase-dependent downregulation of MRE11
Source: Oncogene. 2017 Oct 2;37(4):427–38. doi: 10.1038/onc.2017.340 (PMC5799716; doi:10.1038/onc.2017.340)

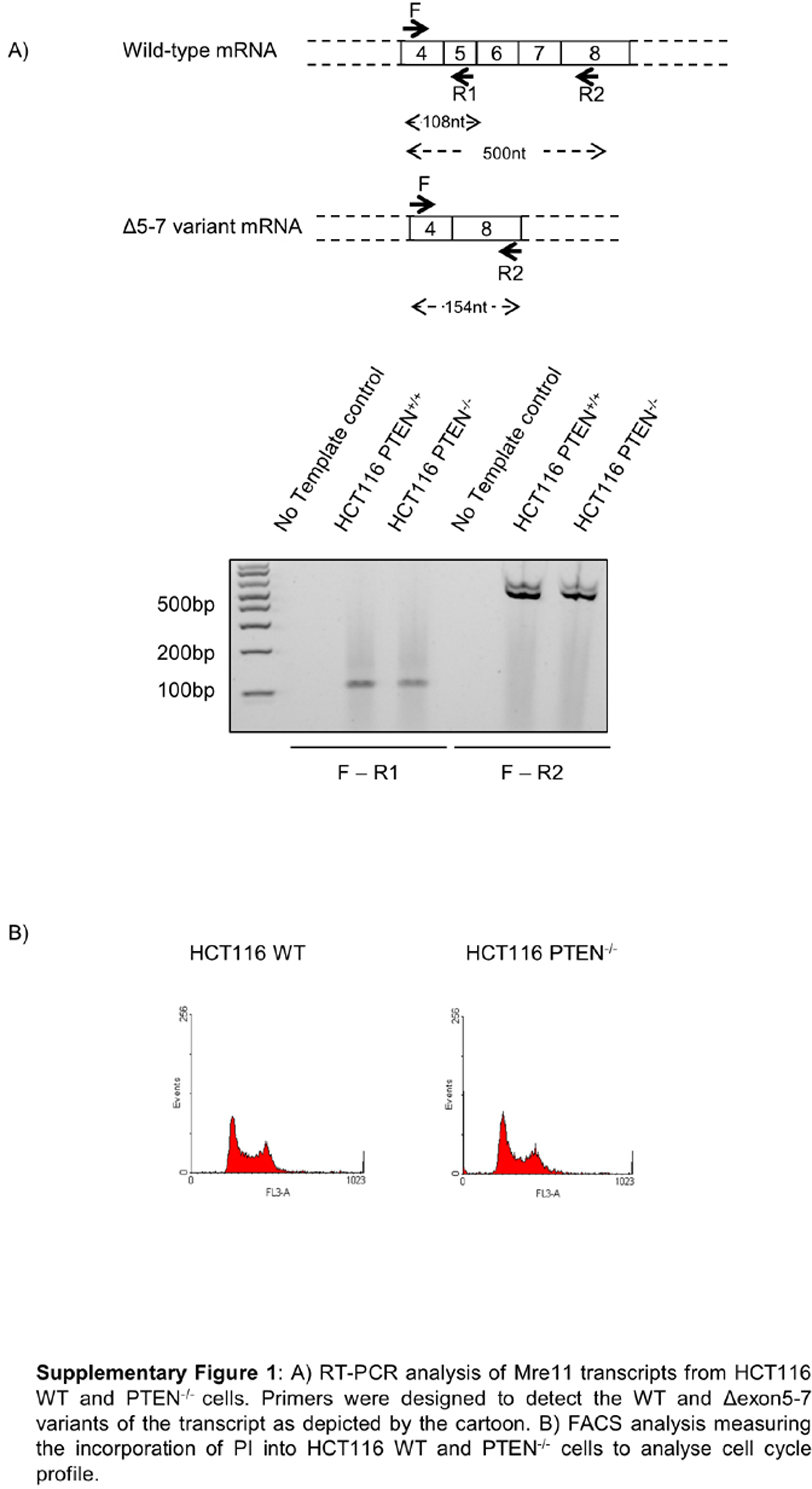

Supplement: Supplementary Figure 1 [file onc2017340x1.tif]

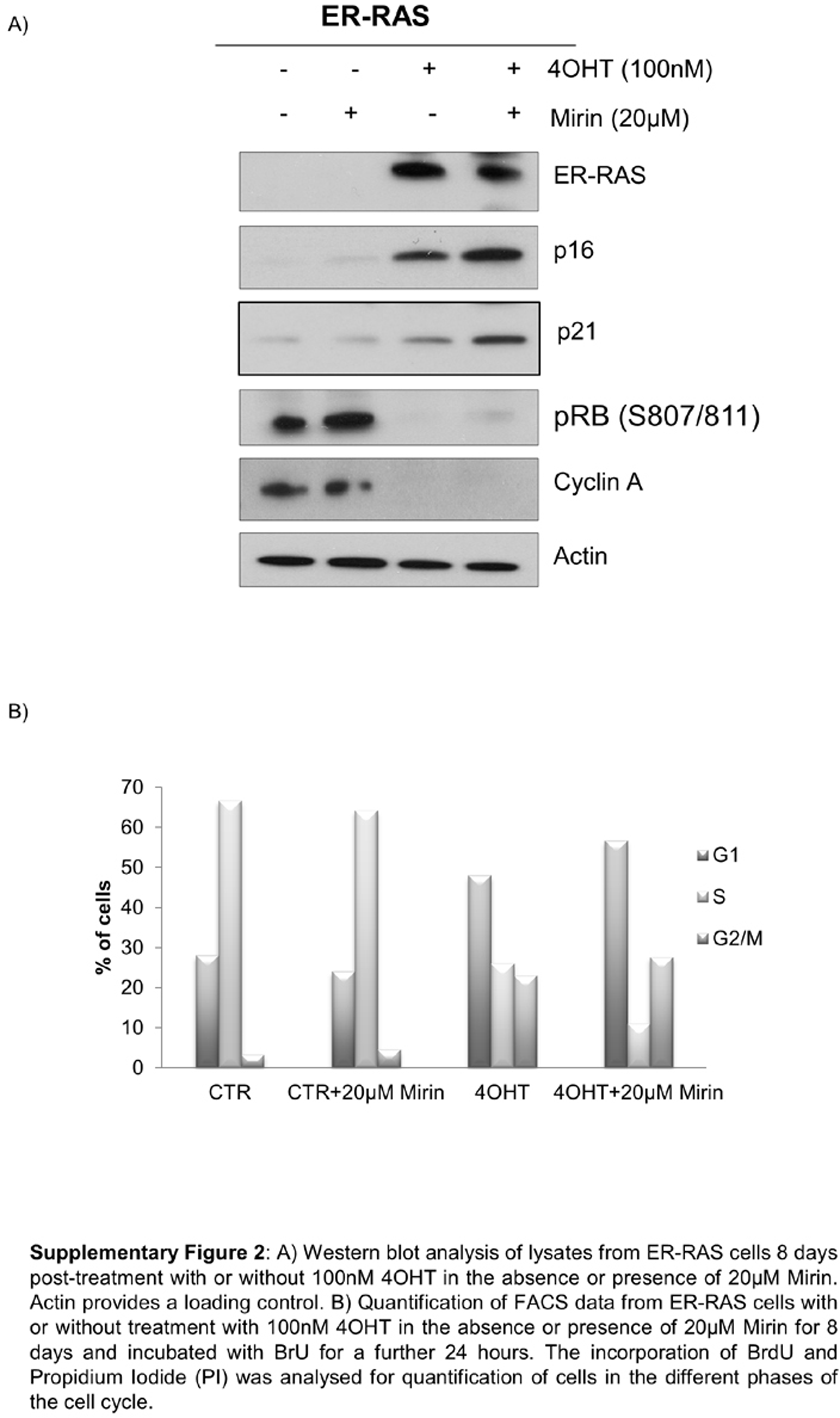

Supplement: Supplementary Figure 2 [file onc2017340x2.tif]
