# Supplementary material for: AKT overactivation can suppress DNA repair via p70S6 kinase-dependent downregulation of MRE11
Source: Oncogene. 2017 Oct 2;37(4):427–38. doi: 10.1038/onc.2017.340 (PMC5799716; doi:10.1038/onc.2017.340)
Supplement: Supplementary Tables [file onc2017340x3.pdf]

Supplementary Table 1: List of primers used for RT-PCR analysis.

**Gene name**		**Primers**
Mre11	F<br>R	GCCTTCCCGAAATGTCACTA<br>TTCAAAATCAACCCCTTTCG
Rad50	F<br>R	CTTGGATATGCGAGGACGAT<br>CCAGAAGCTGGAAGTTACGC
Nbs1	F<br>R	TTGGTTGCATGCTCTTCTTG<br>GGCTGCTTCTTGGACTCAAC
PTEN	F<br>R	GCAGAAAGACTTGAAGGCGTA<br>AGCTGTGGTGGGTTATGGTC
MRE11 WT	F<br>R	ATTGTATGGGTGATCGGCCT<br>TGTTGAGGTTGCCATCTTGA
MRE11 MUTANT	F<br>R	ATTGTATGGGTGATCGGCCT<br>TCATGTTCATGGCCCCAGAT

Supplementary Table 2: List of antibodies used for Western blotting (WB) or immunofluorescence (IF).

**Antigen**	**Host**	**Dilution**	**Source**	**Assay**
Anti-Flag® M2	Mouse	1:10000	Sigma F1804	WB
Akt (pan)(40D4)	Mouse	1:1000	Cell signalling 2920	WB
Phospho-Akt (Ser473)(D9E) XP®	Rabbit	1:1000	Cell signalling 4060	WB
Beta-actin	Mouse	1:5000	Sigma A1978	WB
Chk1 (2G1D5)	Mouse	1:1000	Cell signalling 2360	WB
Phospho-Chk1 (Ser345)	Rabbit	1:1000	Cell signalling 2341	WB
Chk2 (1C12)	Mouse	1:1000	Cell signalling 3440	WB
Phospho-Chk2 (Thr68)	Rabbit	1:1000	Cell signalling 2661	WB
Cyclin A	Rabbit	1:1000	Santa Cruz SC-751	WB
Phospho-GSK-3α/β (Ser21/9)	Rabbit	1:1000	Cell signalling 9331	WB
Mre11 (C16)	Goat	1:1000	Santa-Cruz 5859	WB
Nibrin (Nbs1)(H300)	Rabbit	1:1000	Santa Cruz 11431	WB
PTEN (D4.3) XP®	Rabbit	1:1000	Cell signalling 9188	WB
Phospho-Rb (Ser807/811)	Rabbit	1:1000	Cell signalling 9308	WB
p21	Mouse	1:1000	Santa Cruz SC-817	WB
p53 (1C12)	Mouse	1:1000	Cell signalling 2524	WB
p53 (Ser15)	Rabbit	1:1000	Cell signalling 9284	WB
p16 (G175-405)	Mouse	1:1000	BD 51-1325GR	WB
Rad50 (13B3/2C6)	Mouse	1:1000	Abcam 89	WB
RAS	Mouse	1:1000	BD 610001	WB
RPA	Rat	1:200	Cell signalling 2208	IF
p70S6K (Thr389)(1A5)	Mouse	1:1000	Cell signalling 9206	WB

S6 Ribosomal protein (Ser240/244)(D68F8) XP®	Rabbit	1:1000	Cell signalling 5364	WB
γ-H2Ax	Mouse	1:200	Millipoore 05-636	IF
